# Mitochondrial uncoupling proteins protect human airway epithelial ciliated cells from oxidative damage

**DOI:** 10.1073/pnas.2318771121

**Published:** 2024-02-28

**Authors:** Akansha Jain, Bo Ram Kim, Wenjie Yu, Thomas O. Moninger, Philip H. Karp, Brett A. Wagner, Michael J. Welsh

**Affiliations:** ^a^Department of Internal Medicine, Pappajohn Biomedical Institute, Roy J. and Lucille A. Carver College of Medicine, University of Iowa, Iowa City, IA 52242; ^b^Department of Molecular Physiology and Biophysics, Pappajohn Biomedical Institute, Roy J. and Lucille A. Carver College of Medicine, University of Iowa, Iowa City, IA 52242; ^c^HHMI, Department of Internal Medicine, University of Iowa, Iowa City, IA 52242; ^d^Free Radical and Radiation Biology Program, Department of Radiation Oncology, Roy J. and Lucille A. Carver College of Medicine, University of Iowa, Iowa City, IA 52242

**Keywords:** reactive oxygen species, oxygen, metabolism, lung, motile cilia

## Abstract

Apical cilia on epithelial cells defend the lung by propelling pathogens and particulates out of the respiratory airways. Ciliated cells produce ATP that powers cilia beating by densely grouping mitochondria just beneath the apical membrane. However, this efficient localization comes at a cost because electrons leaked during oxidative phosphorylation react with molecular oxygen to form superoxide, and thus, the cluster of mitochondria creates a hotspot for oxidant production. The relatively high oxygen concentration overlying airway epithelia further intensifies the risk of generating superoxide. Thus, airway ciliated cells face a unique challenge of producing harmful levels of oxidants. However, surprisingly, highly ciliated epithelia produce less reactive oxygen species (ROS) than epithelia with few ciliated cells. Compared to other airway cell types, ciliated cells express high levels of mitochondrial uncoupling proteins, UCP2 and UCP5. These proteins decrease mitochondrial protonmotive force and thereby reduce production of ROS. As a result, lipid peroxidation, a marker of oxidant injury, decreases. However, mitochondrial uncoupling proteins exact a price for decreasing oxidant production; they decrease the fraction of mitochondrial respiration that generates ATP. These findings indicate that ciliated cells sacrifice mitochondrial efficiency in exchange for safety from damaging oxidation. Employing uncoupling proteins to prevent oxidant production, instead of relying solely on antioxidants to decrease postproduction oxidant levels, may offer an advantage for targeting a local area of intense ROS generation.

## Significance

Motile cilia protruding from airway epithelial cells propel pathogens out of the lungs. Respiratory ciliated cells have an efficient supply chain that provides ATP to power cilia beating; the producers of ATP (mitochondria) are clustered just beneath the consumers (cilia) and an abundant supply (oxygen) in air covering the cells. But a byproduct of this organization, reactive oxygen species (ROS), pose the risk of injury. Human airway ciliated cells balance the requirement for energy and the potential for oxidant injury with mitochondrial uncoupling proteins, which decrease mitochondrial efficiency but minimize ROS production. Improved understanding of airway metabolism may yield benefit for people challenged by localized hyperoxia because of treatment with inhaled oxygen.

Epithelia lining the respiratory airways provide the first line of defense for the lung (1). Airway mucus traps inhaled pathogens and particulate material, and beating cilia propel the mucus out of the lung by mucociliary transport (2–4). Cilia beating is driven by dynein motor proteins that are powered by ATP hydrolysis (5–7). Feeding ATP to cilia likely consumes a major fraction of the ATP produced by ciliated airway epithelial cells.^*^ Ciliated cells solve the challenge of supplying ATP to cilia by grouping together many mitochondria in a relatively tight cluster located just beneath the apical membrane (8). ATP they produce can then diffuse up into apical cilia (9).

The strategy of packing many mitochondria together in a small volume introduces the risk of oxidant injury. That is because mitochondria produce ATP through oxidative phosphorylation, which is not entirely efficient. The mitochondrial electron transport chain (ETC) generates an electrochemical gradient by pairing the movement of electrons to the transfer of protons out of the mitochondrial matrix; O_2_ is the final electron acceptor of the ETC (10, 11). This activity generates a protonmotive force that the mitochondrial ATP synthase uses to generate ATP. However, the ETC leaks electrons (12–16). Those electrons can then react nonenzymatically with molecular O_2_ to form superoxide (O_2_^·−^); estimates of O_2_^·−^ produced vary from 0.2 to 2% of the O_2_ consumed (17). O_2_^·−^ can then convert to hydrogen peroxide and other reactive oxygen species (ROS) (12–15, 17). Because each mitochondrion leaks electrons and produces O_2_^·−^, clustering mitochondria together creates a potential hotspot for ROS production.

Proximity to O_2_ in air intensifies the risk of mitochondrial ROS generation. Whereas most cells in the body are exposed to ~2 to 7% O_2_ (18, 19), airway epithelia with its thin layer of liquid are covered by much higher O_2_ levels (~18.5%).^†^ Additionally, the higher the O_2_ concentration, the greater the mitochondrial O_2_^·−^ production (12^‡^) Although ROS are essential for cell signaling (20), too much ROS is pathogenic, causing mutations in mitochondrial and nuclear DNA, lipid peroxidation of cellular membranes, and protein oxidation that impairs enzymatic processes (12, 14, 15, 21).

Thus, ciliated airway cells face a unique challenge from ROS production. We hypothesized that airway ciliated cells either have abnormally high ROS production, or they have developed ways to minimize ROS levels. When we found an inverse relationship between the abundance of ciliated cells and ROS levels, we pursued the second alternative.

## Results

### Airway Ciliated Cells Contain a Dense Subapical Population of Mitochondria that Are Required for Ciliary Beating.

**Fig. 1.**
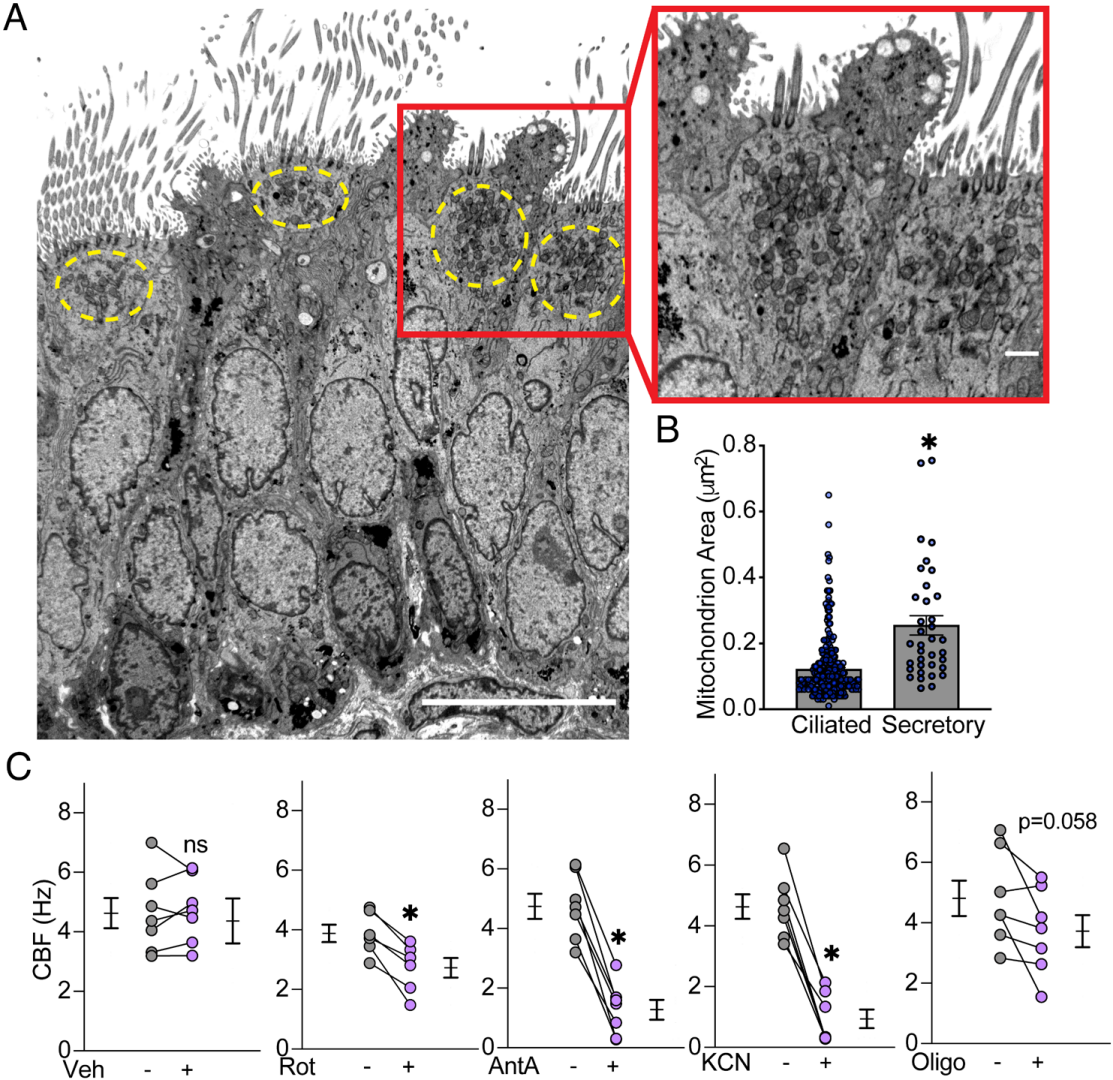
Mitochondria are located apically in airway ciliated cells and power cilia beating. (*A*) Transmission electron micrograph of airway epithelia showing ciliated and secretory/goblet cells. The yellow dashed circle outlines mitochondria located directly below cilia. The scale bar indicates 10 µm. The *Inset* shows apical mitochondria. The scale bar indicates 1 µm. (*B*) Quantification of the mitochondria area in ciliated and secretory cells. Bars show mean ± SEM. The asterisk indicates *P* < 0.0001 by Student’s *t* test. (*C*) Ciliary beat frequency (CBF) in the presence of the indicated inhibitors of the electron transport chain compared to vehicle control. CBF measurements were performed at 20°C. Each set of data points and lines is from a different human donor. Bars and whiskers indicate mean ± SEM. The asterisk indicates *P* < 0.01 by paired Student’s *t* test.

We tested mitochondrial localization in human airway epithelia by immunostaining translocases of the outer mitochondrial membrane 20 and 70 (TOM20, TOM70). Mitochondria staining was predominantly apical in acetylated α-tubulin or β-tubulin IV positive human and pig ciliated cells (*SI Appendix*, Fig. S1 *A*–*C*). Transmission electron microscopy (TEM) of human airways extended the immunofluorescence data, revealing dense apical clusters of mitochondria in ciliated but not the neighboring goblet/secretory cells (Fig. 1*A*). TEM also uncovered differences in mitochondrial size; ciliated cell mitochondria were smaller than those in goblet/secretory cells (Fig. 1*B*). These data are consistent with previous findings that showed bunched, apical mitochondria in ciliated airway cells (8).

To test the prediction that mitochondria fuel cilia beating, we inhibited several ETC complexes and measured CBF. Applying inhibitors of complex I (rotenone), complex III (antimycin A), complex IV (cyanide), and ATP synthase (oligomycin) acutely decreased CBF (Fig. 1*C*). These results confirm and extend an earlier report that antimycin A decreased CBF (22).

The distinct localization and size of ciliated cell mitochondria led us to ask whether their function might differ from that of other airway epithelial cells, especially in ROS production.

### Highly Ciliated Epithelia Have Decreased Intracellular ROS Levels.

**Fig. 2.**
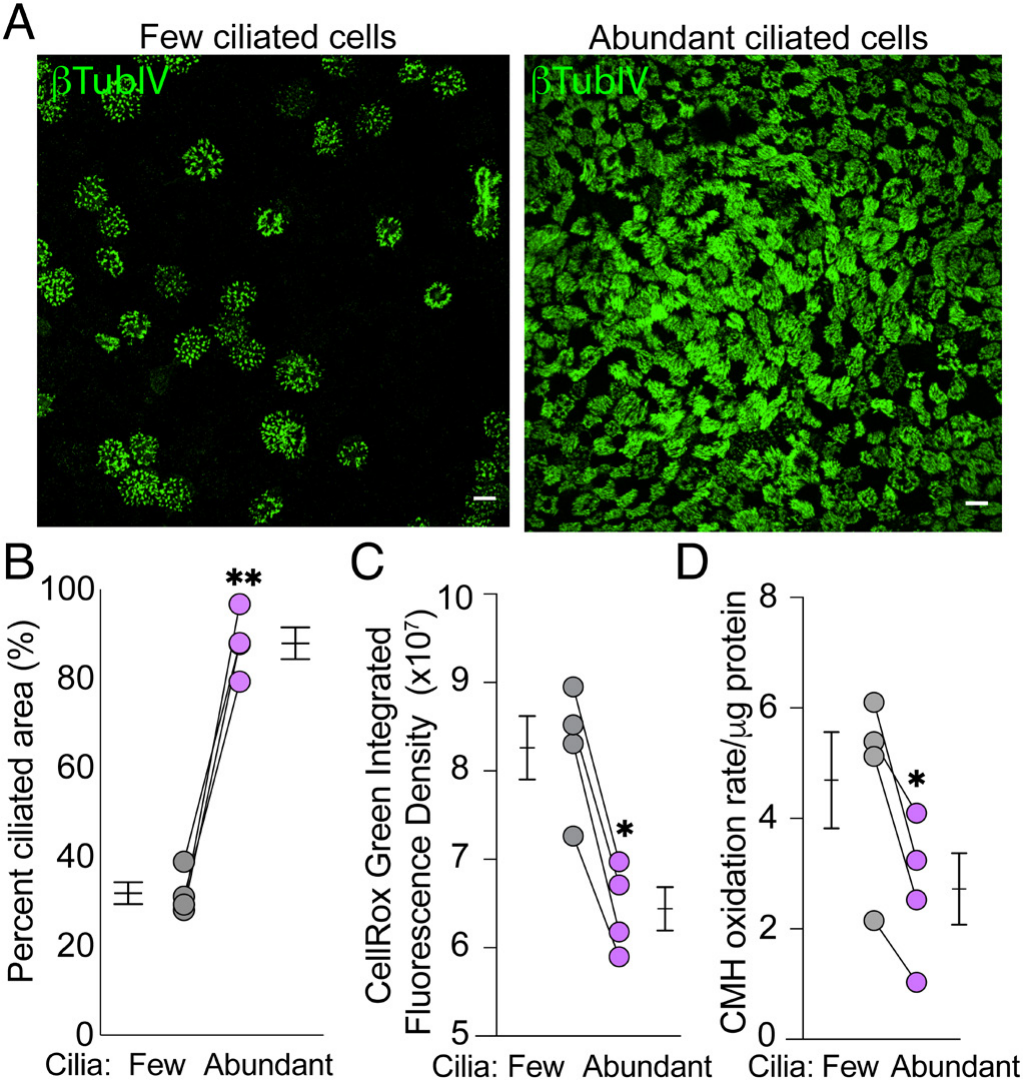
Airway epithelia with abundant ciliated cells produce less ROS than epithelia with few ciliated cells. (*A*) Immunofluorescence images showing differentiated human airway epithelia with few and abundant ciliated cells (grown in USG media vs. Pneumacult ALI media, Method 1). β-tubulin IV immunostaining marks cilia (green). The scale bar indicates 10 µm. (*B*) Quantification of the percentage of airway surface covered by cilia determined by immunostaining with β-tubulin IV. (*C* and *D*) Levels of ROS in epithelia with few vs. abundant ciliation measured using CellROX-Green fluorescence assay (*C*) and ESR (*D*).

We tested the hypothesis that epithelia with abundant ciliated cells would generate more ROS. To vary ciliated cell numbers, we differentiated human airway epithelial cells at the air–liquid interface in either USG medium or Pneumacult-ALI medium (Method 1). This produced epithelial with few vs. abundant cilia, respectively (Fig. 2 *A* and *B*). Of note, epithelia differentiated in USG medium appeared slightly larger. We studied all epithelia at 18.5% O_2_. To measure intracellular ROS levels, we used CellROX-green, a fluorescent probe that detects hydroxyl radicals and superoxide anions (23, 24). Surprisingly, the data indicated that epithelia with more ciliated cells had lower ROS levels (Fig. 2*C*). To confirm this unexpected finding, we measured intracellular oxidation of the cell permeable spin probe 1-hydroxy-3-methoxycarbonyl-2,2,5,5-tetramethylpyrrolidine (CMH) with electron spin resonance (ESR) (25). CMH can be oxidized by intracellular oxidants allowing for quantitative detection of stable nitroxide (25). The outcome was similar to that obtained with a fluorescent probe (Fig. 2*D*). As an additional means of generating epithelia with variable ciliation, we differentiated cells in Pneumacult-ALI medium at either 0.5% O_2_ or 18.5% O_2_ (Method 2), this generates epithelia with few vs. abundant cilia, respectively (*SI Appendix*, Fig. S2*A*) (26). We then studied them at 18.5% O_2_. Epithelia with more abundant cilia produced less intercellular ROS (*SI Appendix*, Fig. S2*B*).

These results indicated that highly ciliated epithelia had lower intracellular ROS levels compared to less ciliated epithelia. These findings suggested that ciliated cells either scavenge more ROS or produce less ROS than other cells in the epithelium.

### Ciliated Cells Have Variable Levels of Antioxidant Transcripts.

**Fig. 3.**
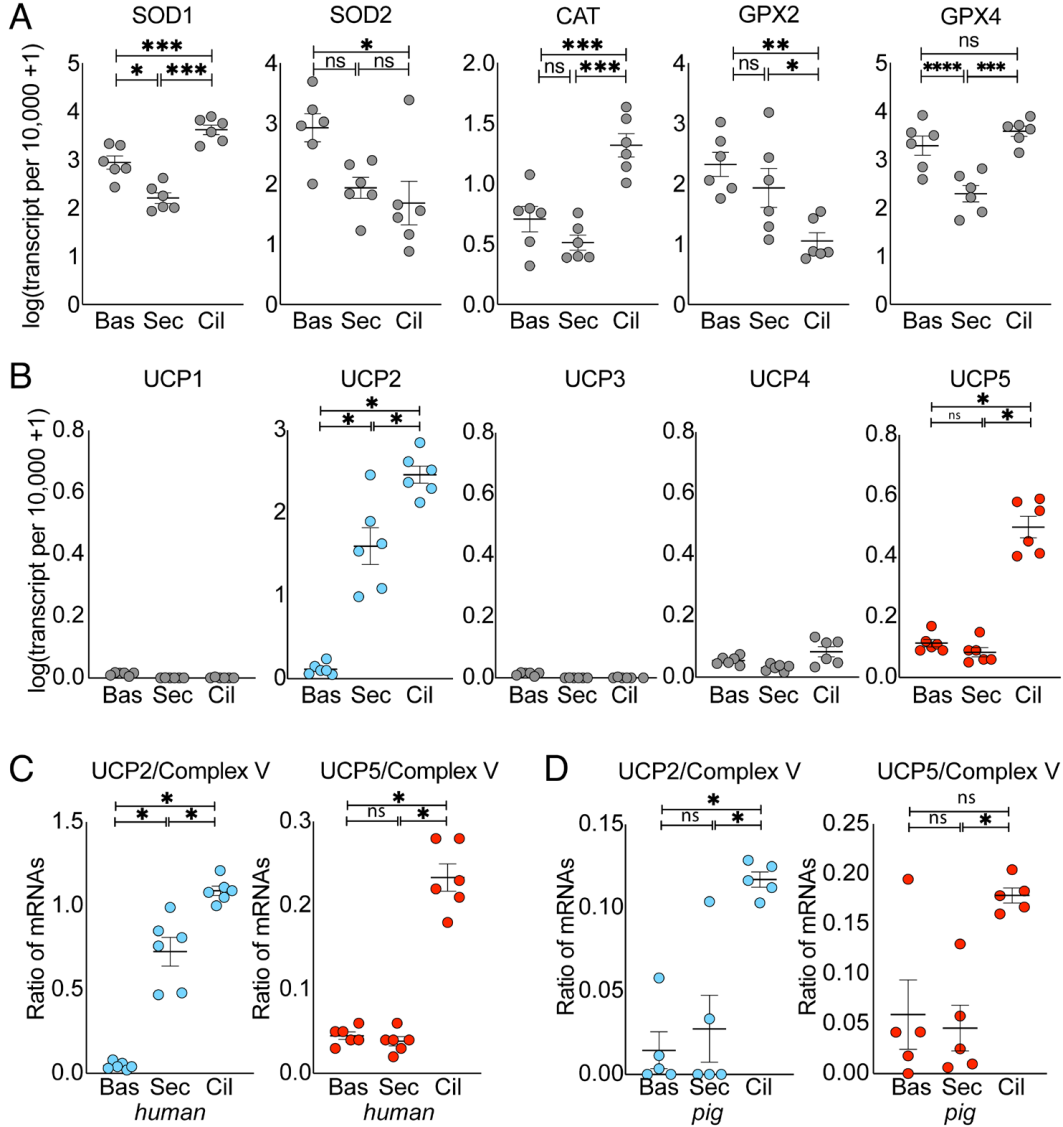
Airway ciliated cells express mitochondrial uncoupling proteins UCP2 and UCP5. (*A*) mRNA levels of indicated antioxidant enzymes in basal (Bas), secretory (Sec), and ciliated (Cil) cells of large airway tissue. Data are from a public single-cell mRNA database of human large airways (27). (*B*) Expression of *UCP1-UCP5* mRNAs in airway epithelial cells from the same database as in panel *A*. (*C*) Ratios of *UCP2* and *UCP5* mRNA levels to the average of multiple Complex V mRNA levels of the electron transport chain (ETC). Data are from the same database as in panel *A*. (*D*) Ratios *UCP2* and *UCP5* mRNA levels to the average of mRNA levels of multiple Complex V proteins from a public single-cell mRNA database of pig large airways (29). In panels *A*–*D*, each data point is from a different human donor or pig. Bars and whiskers indicate mean ± SEM. Asterisks indicate *P < 0.05, **P < 0.01, and ***P < 0.001 by ANOVA.

As an indication of the potential for scavenging ROS, we interrogated published single-cell RNA sequencing (scRNA-seq) databases of human airway tissue (27, 28). We compared transcript levels of antioxidants in ciliated cells vs. the two other abundant cell types in airway epithelia, goblet/secretory cells, and basal cells. Superoxide dismutase (SOD) converts O_2_^·−^ to hydrogen peroxide (H_2_O_2_). Cytosolic SOD1 transcript levels were higher, but mitochondrial SOD2 levels were lower in ciliated cells compared to the other cell types (Fig. 3*A* and *SI Appendix*, Fig. S2*C*). Catalase (CAT) converts H_2_O_2_ to water and O_2_. CAT levels were higher in ciliated cells. Glutathione peroxidases (GPX) reduce H_2_O_2_ to water and lipid hydroperoxides to alcohols. Of the two most abundant GPX transcripts, GPX2 levels were lower in ciliated cells, and GPX4 mRNA levels were higher in ciliated cells than in goblet/secretory cells but the same as in basal cells. These results suggest that antioxidants likely play a role in protecting ciliated cells from ROS-induced damage. However, there was not a consistent pattern of mRNA differences for antioxidants in ciliated vs. other airway epithelial cells, and the maximum differences were twofold or less. Therefore, we turned our attention to mechanisms that might decrease ROS production.

### Ciliated Cell Mitochondria Are Equipped with UCP2 and UCP5.

To investigate the possibility that ROS production might be decreased in ciliated cells, we asked whether they express uncoupling proteins (UCPs). UCPs are inner mitochondrial membrane proteins that allow protons to leak back into the matrix, thereby dissipating the protonmotive force. Thus, they uncouple electron transport from ATP synthesis, decrease the protonmotive force, and thereby decrease mitochondrial ROS production (12). These proteins have been studied extensively in brown adipose tissue (UCP1), muscle (UCP3), and the central nervous system (UCP4 and UCP5) (30–32).

We probed published scRNA-seq databases and found that *UCP2* and *UCP5* transcripts were significantly higher in ciliated cells than in goblet/secretory or basal cells (Fig. 3*B* and *SI Appendix*, Fig. S2*D*) (27, 28). To control for differences in mitochondrial content, we also normalized levels of *UCP2* and *UCP5* transcripts to transcript levels of oxidative phosphorylation proteins in complexes I-V; *UCP2* and *UCP5* mRNAs remained enriched in ciliated cells vs. other cell types (Fig. 3*C* and *SI Appendix*, Fig. S2*E*). The same pattern was seen in a published scRNA-seq database from pig airway epithelia, suggesting that UCP expression is conserved across species (Fig. 3*D*) (29).

**Fig. 4.**
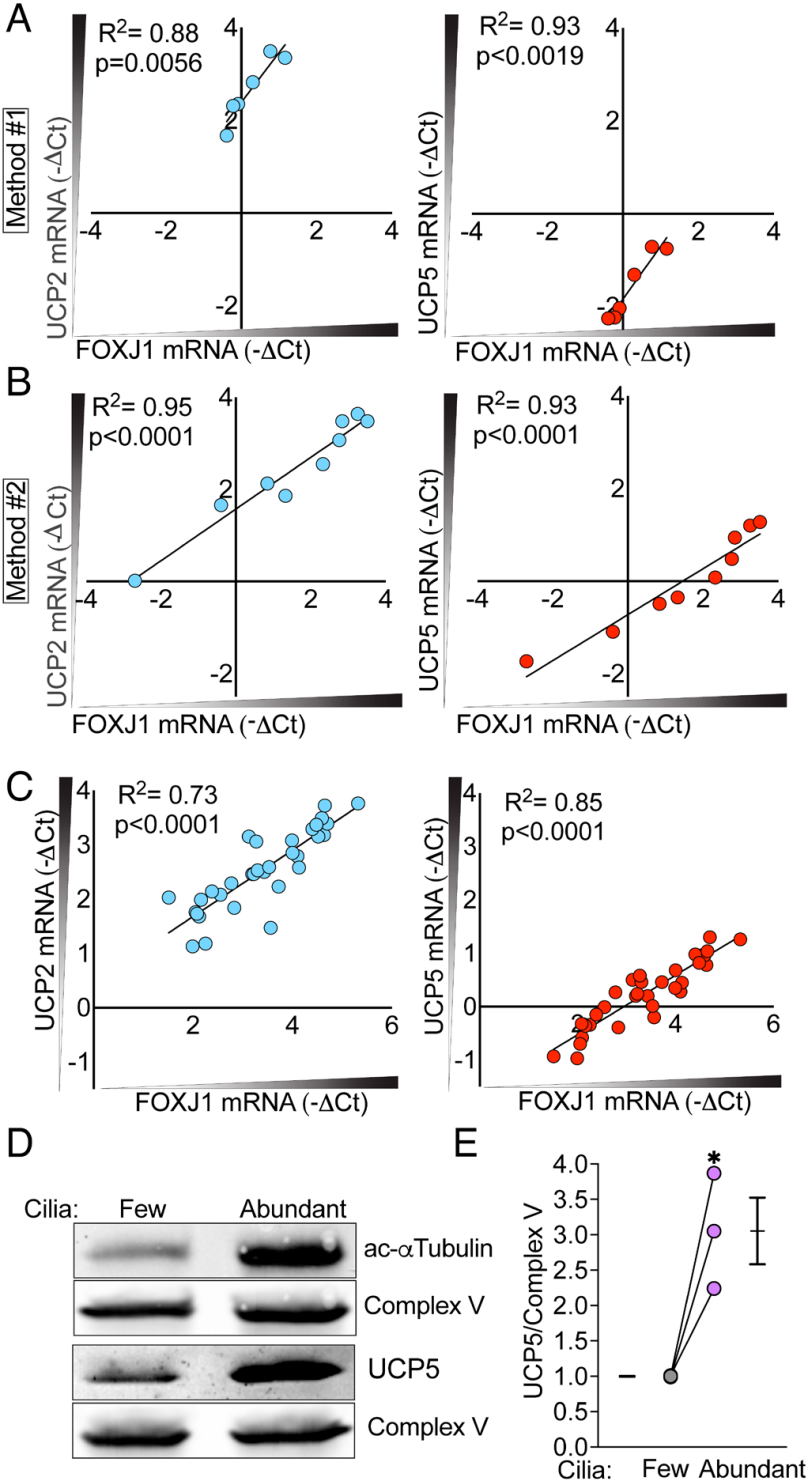
UCP2 and UCP5 expression are directly related to ciliation of human airway epithelia. (*A*) RT-qPCR data showing relationships of *UCP2* and *UPC5* mRNA levels to *FOXJ1* mRNA levels (indicating ciliated cells) in cultures of human airway epithelia. Epithelial cells were differentiated in USG vs. Pneumacult-ALI media (Method 1). (*B*) Methods were the same as in panel A except that epithelial cells were differentiated in Pneumacult-ALI media at either 0.5% vs. 18.5% O_2_ (Method 2). (*C*) RT-qPCR data showing relationships of *UCP2* and *UPC5* mRNA levels to *FOXJ1* mRNA. All epithelia were differentiated in USG media at 18.5% O_2_, and variations are donor-to-donor differences. For panels *A*–*C*, each data point is from epithelia from a different human donor. Lines are linear least squares fit to the data; R^2^ and *P* values are shown. (*D*) Immunoblot showing acetylated α-tubulin protein and UCP5 protein in epithelia with few vs. abundant ciliated cells (differentiated in USG vs. Pneumacult-ALI media, Method 1). Complex V of the ETC was used as the protein loading control. (*E*) Quantification of the UCP5/Complex V ratio from western blots like that shown in panel *D*. Each data point is from a different human donor. Bars and whiskers indicate mean ± SEM. The asterisk indicates *P* < 0.05 by paired Student’s *t* test.

To validate the scRNA-seq data, we performed qRT-PCR on epithelia with few vs. abundant cilia, that is, epithelia cultured in USG medium vs. Pneumacult-ALI medium (Method 1), respectively. We compared the mRNA abundance of *UCP2* and *UCP5* vs. the abundance of mRNA for *FOXJ1*; *FOXJ1* is a marker of ciliated cells (33). As *FOXJ1* mRNA increased, so did *UCP2* and *UCP5* mRNA (Fig. 4*A*). As an additional test, we differentiated cells in Pneumacult-ALI medium at either 0.5% O_2_ or 18.5% O_2_, (Method 2) (26), and then studied them at 18.5% O_2_. Epithelia with more ciliated cells, as indicated by greater *FOXJ1* mRNA, had a greater abundance of *UCP2* and *UCP5* mRNA (Fig. 4*B*). Because the difference in culture methods (USG vs. Pneumacult-ALI media and differentiation in 0.5% O_2_ vs. 18.5% O_2_) could potentially influence *UCP* expression independently of ciliation, we also took advantage of donor-to-donor variability in the degree of ciliation. Using epithelia from multiple donors cultured in USG media at 18.5% O_2_, *UCP2* and *UCP5* mRNA levels correlated directly with *FOXJ1* mRNA levels (Fig. 4*C*). As a control, mRNA levels for mitochondrial complex proteins did not correlate with the abundance of *FOXJ1* mRNA (*SI Appendix*, Fig. S2*F*). Western blots also showed increased amounts of UCP5 relative to a mitochondrial protein in epithelia with abundant vs. few cilia (Fig. 4 *D* and *E*). Despite attempts with multiple antibodies, western blots for UCP2 were not successful.

**Fig. 5.**
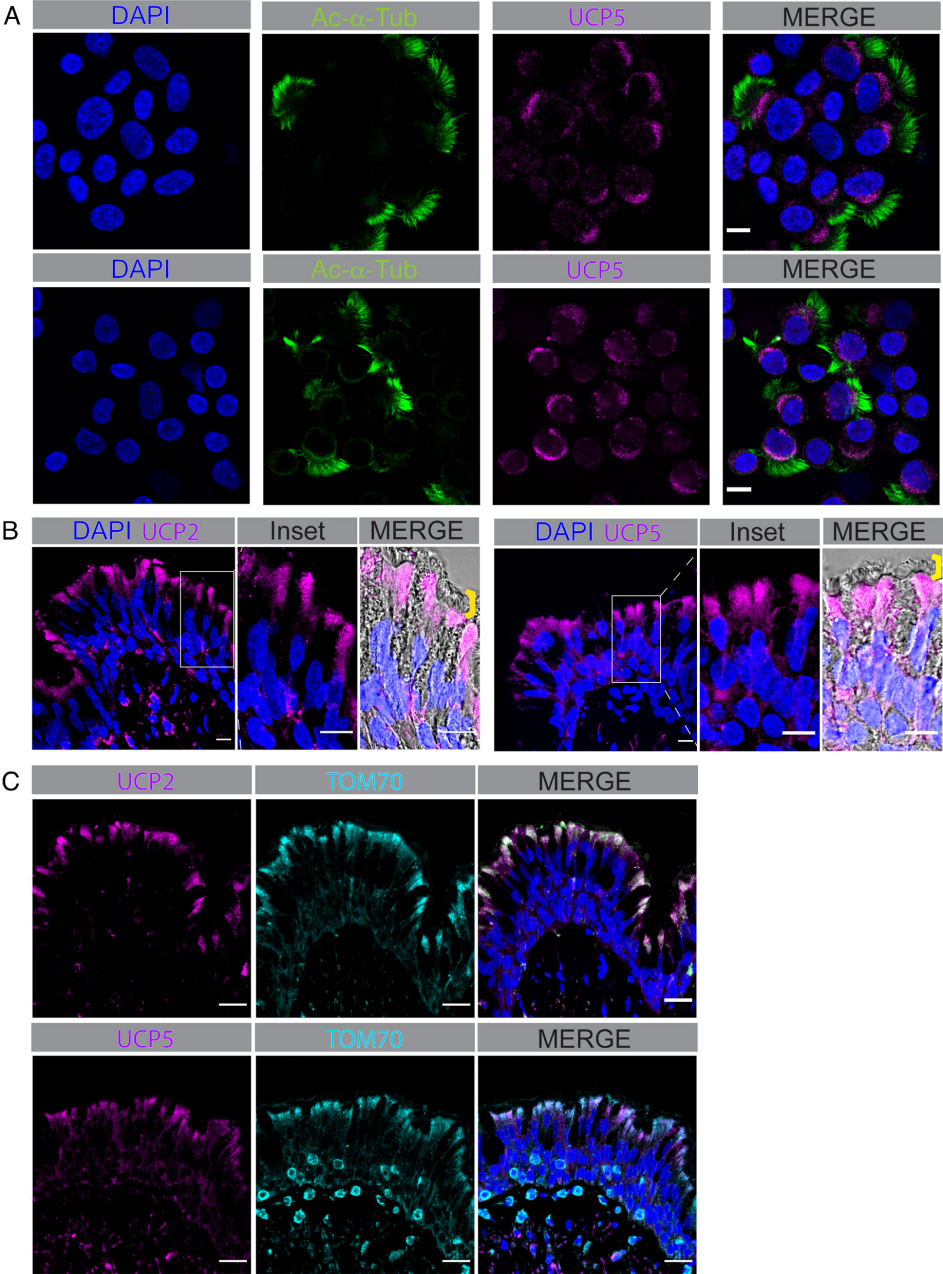
UCP2 and UCP5 are located just beneath the cilia in ciliated airway epithelial cells. (*A*) Immunofluorescence images showing acetylated α-tubulin marking cilia (green), UCP2 (*Top*) and UPC5 (*Bottom*) (magenta), and DAPI marking nuclei (blue) in dissociated human airway epithelial cells. Scale bars indicate 10 µm. (*B*) Immunofluorescence images showing UCP2 (*Left*) and UPC5 (*Right*) (magenta) and DAPI marking nuclei (blue) in human large airway tissue. Scale bars indicate 100 µm. Merged panels include transmitted light marking tissue edge and ciliated cells marked with a yellow line. Scale bars indicate 10 µm. (*C*) Immunofluorescence images showing colocalization of UCP2 (*Top*) and UPC5 (*Bottom*) (magenta) and TOM70 marking mitochondria (magenta) in human large airway tissue. Merged panels include DAPI. Scale bars indicate 100 µm.

To further test whether ciliated cells are the specific cells expressing UCP2 and UCP5, we immunostained dissociated cells and found both proteins in ciliated cells but not in other cell types of cultured airway epithelia (Fig. 5*A*). In addition, both UCPs localized beneath the apical membrane, the location where mitochondria cluster. We observed the same expression pattern and localization in tissue from human airways (Fig. 5*B*). UCP2 and UCP5 colocalized with TOM70 (Fig. 5*C*). The presence of UCPs suggested that they would uncouple electron transport from ATP synthesis in ciliated airway cells.

### Airway Epithelia with Abundant Cilia Exhibit Uncoupled Mitochondrial Respiration.

**Fig. 6.**
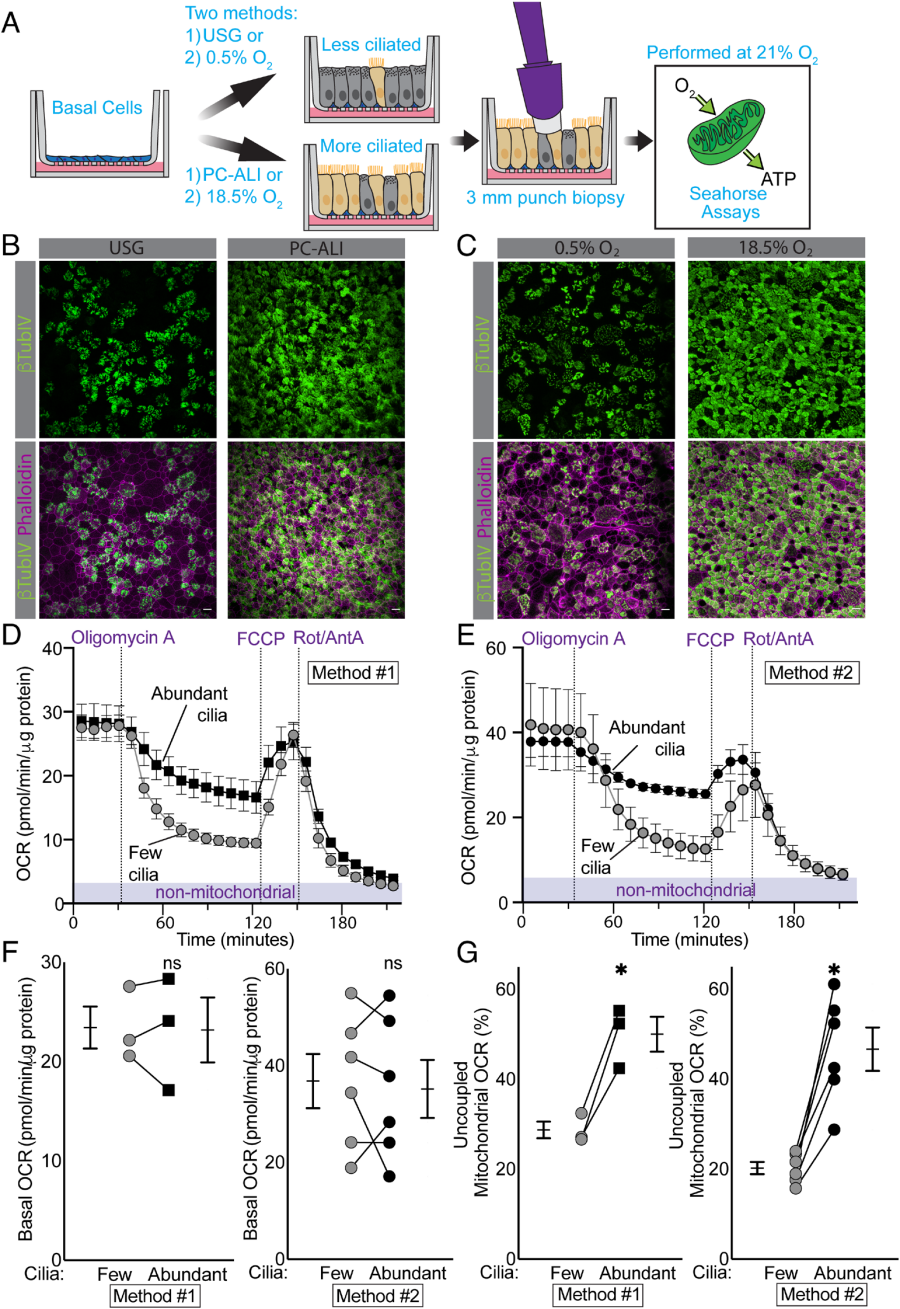
Epithelia with abundant ciliated cells have high levels of uncoupled mitochondrial respiration. (*A*) Schematic showing the strategy for differentiating cultures with few vs. abundant ciliated cells, sampling using 3-mm punches of epithelia, and measuring O_2_ consumption. Studies were performed at 18.5% O_2_. (*B*) Immunofluorescence images showing acetylated α-tubulin marking cilia (green) and actin staining (magenta) marking cell boundaries. Epithelia were grown in USG (*Left*) vs. Pneumacult-ALI (PC-ALI) (*Right*) (Method 1). Scale bars indicate 10 µm. (*C*) Same as Panel *B* except that epithelia were grown under 0.5% O_2_ (*Left*) vs. 18.5% O_2_ (*Right*); (Method 2). (*D*) Representative mitochondrial stress test showing responses after injection of oligomycin A (3 μM), FCCP (0.5 μM), and rotenone (1 μM) and antimycin A (1 μM) for epithelia differentiated under Method 1. Data are mean ± SEM of three to four technical replicates. (*E*) Same as Panel *D* except that epithelia were differentiated with Method 2. (*F*) Basal OCR in epithelia with few and abundant ciliated cells (Methods 1 and 2). (*G*) Uncoupled respiration as a percentage of total mitochondrial respiration in epithelia with few and abundant ciliated cells (Methods 1 and 2). For panels *F* and *G*, each set of data points and lines is from a different human donor. Bars and whiskers indicate mean ± SEM. The asterisk indicates *P* < 0.05 by paired Student’s *t* test.

To test the prediction that ciliated cells have a greater fraction of respiration that is not linked to ATP production, we measured mitochondrial oxygen consumption rates (OCR) (Fig. 6*A*). We used epithelia differentiated in different media (Method 1) or different O_2_ (Method 2) to generate epithelia with few and abundant cilia (Fig. 6 *B* and *C*) (26). Basal respiration was the same regardless of the degree of ciliation (Fig. 6 *D*–*F*). When we inhibited ATP synthase with oligomycin, OCR fell, but the decrease was less in epithelia with abundant cilia (Fig. 6 *D* and *E*). We calculated uncoupled respiration as OCR in the presence of oligomycin minus nonmitochondrial OCR (after mitochondrial respiration was inhibited by rotenone+antimycin A). Uncoupled respiration approximately doubled in epithelia with abundant cilia (Fig. 6*G*). These data together with the expression data suggest that UCP2 and UCP5 increased mitochondrial uncoupling in ciliated cells.

### *UCP2* and *UCP5* Knockdown Decreased Proton Leak and Increased Mitochondrial Membrane Potential.

**Fig. 7.**
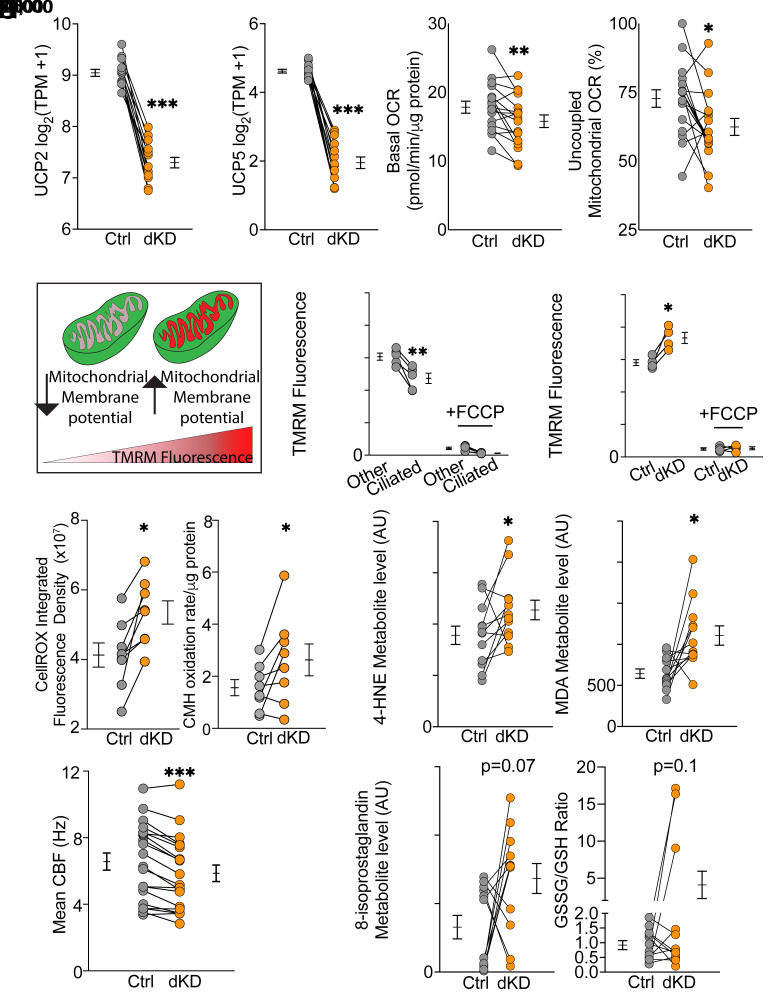
Knockdown of *UCP2* and *UCP5* increased mitochondrial membrane potential, ROS production, and metabolic markers of lipid peroxidation. (*A*) *UCP2* and *UCP5* mRNA levels after control antisense oligonucleotides (Ctrl) and double knockdown (dKD) of *UCP2* and *UCP5*. (*B*) Basal respiration by Ctrl and dKD treated airway epithelia. (*C*) Uncoupled respiration as a percentage of total mitochondrial respiration in Ctrl vs. dKD epithelia. (*D*) Schematic showing that a high mitochondrial membrane potential increases TMRM fluorescence. (*E*) TMRM fluorescence intensity of ciliated cells vs. all other cells (including basal, secretory, and rare cell types) assayed by flow cytometry. The uncoupling agent, FCCP served as a positive control. (*F*) TMRM fluorescence intensity of ciliated cells in Ctrl vs. *UCP2* and *UCP5* dKD treated ciliated cells assayed by flow cytometry. (*G*) Levels of ROS in Ctrl and dKD treated cells, as measured using CellRox-green (*Left*) and ESR (*Right*). ESR rates are normalized to time zero. (*H*) Levels of metabolic markers of lipid peroxidation (4-HNE, MDA, and 8-isoprostaglandin) in Ctrl vs. dKD treated epithelia. Ratios of oxidized to reduced glutathione (GSSG/GSH) in Ctrl vs. dKD treated epithelia. (*I*) CBF in Ctrl vs. dKD treated epithelia. Studies were done at 20 °C. In all panels, each set of data points and lines is from a different human donor. Asterisks indicate ***P < 0.001, **P < 0.01, and *P < 0.05 by paired Student’s *t* test. Bars and whiskers indicate mean ± SEM.

To test the hypothesis that UCP2 and UCP5 increase uncoupling, we knocked down their mRNA using antisense oligonucleotides. Knocking down a single *UCP2* or *UCP5* mRNA did not significantly decrease uncoupled mitochondrial respiration (*SI Appendix*, Fig. S3 *A* and *B*). Therefore, we simultaneously knocked down both *UCP2* and *UCP5* (Fig. 7*A* and *SI Appendix*, Fig. S3*C*). Double knockdown decreased basal OCR, and it decreased uncoupled respiration (Fig. 7 *B* and *C*). These data suggest that UCP2 and UCP5 control the fraction of uncoupled mitochondrial respiration in ciliated cells.

Because UCPs induce a mitochondrial proton leak, we predicted that UCP2 and UCP5 would decrease relative mitochondrial membrane potential in ciliated cells. To test this assessment, we used trimethylrhodamine (TMRM), a membrane potential-dependent fluorescent probe to measure relative mitochondrial membrane potential, an anti-ACE2 antibody to identify ciliated cells, and flow cytometry to report fluorescence (Fig. 7*D*). We found that ciliated cells had a lower mean TMRM fluorescence than nonciliated cells (Fig. 7*E*). FCCP used as a positive control completely dissipated the electrochemical gradient. To confirm that UCP2 and UCP5 were responsible for the decreased mitochondrial membrane potential, we repeated the measurements after knocking down *UCP2* and *UCP5* and found that TMRM fluorescence increased (Fig. 7*F*). These data suggest that UCP2 and UCP5 lower the protonmotive force in ciliated cell mitochondria.

### *UCP2* and *UCP5* Knockdown Increases Intracellular ROS Levels and Lipid Peroxidation.

By introducing a proton leak and decreasing mitochondrial membrane potential, UCP2 and UCP5 should decrease ROS production. Consistent with this prediction, when we knocked down *UCP2* and *UCP5*, ROS increased as measured by a fluorescent probe and by ESR (Fig. 7*G*).

Although ROS have important signaling functions, increased levels can perturb redox homeostasis and cause toxicity (34–36). To assess this possibility, we did several studies using liquid chromatography-mass spectrometry. We found that knocking down *UCP2* and *UCP5* increased markers of lipid peroxidation including the reactive aldehyde 4-hydroxynonenal (4-HNE) and malondialdehyde (MDA) (Fig. 7*H*). There were also nonstatistically significant trends for an increase in 8-isoProstaglandin F2α (8-iso-PGF2α) and the ratio of oxidized to reduced glutathione (GSSG/GSH) with donor-dependent variation. Previous studies reported that high ROS levels decrease CBF (37), and we found that *UCP2* and *UCP5* knockdown decreased CBF (Fig. 7*I*). These findings suggest that UCP2 and UCP5 may decrease accumulation of toxic lipid peroxidation byproducts and thereby maintain ciliated cell function.

## Discussion

**Fig. 8.**
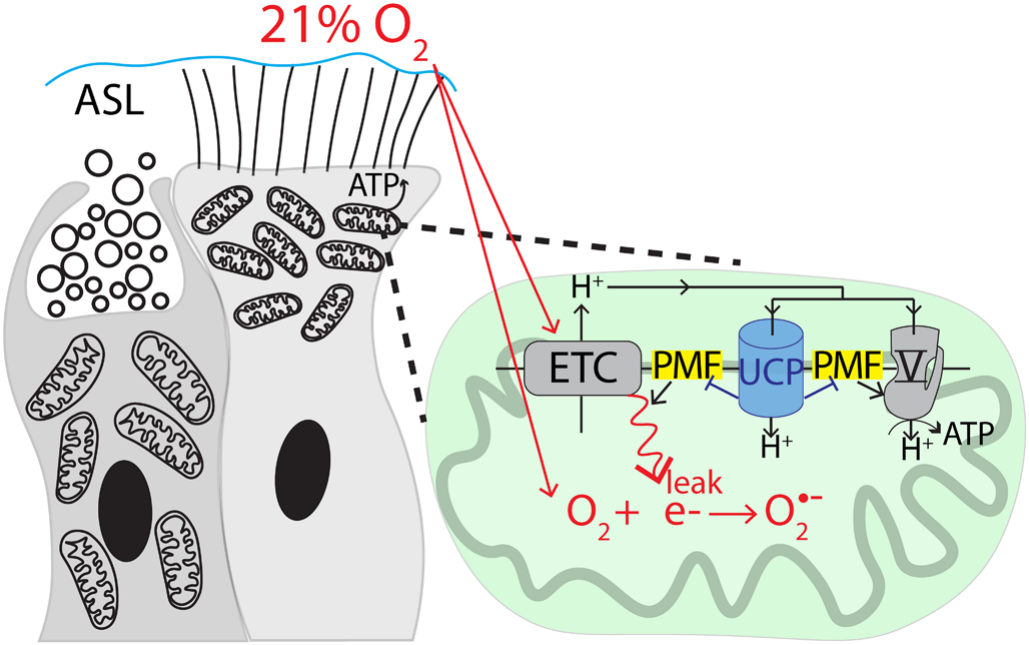
Model of ciliated cell mitochondrial function. Clustering of mitochondria and the relatively high O_2_ levels make the apical region of ciliated cells a hotspot for ROS production. UCP2 and UCP5 on the inner mitochondrial membrane decrease the protonmotive force, thereby balancing mitochondrial efficiency with decreased ROS production.

Airway ciliated cells face a unique challenge from abnormally high ROS production due to tight subapical clustering of mitochondria, which leak electrons from ETC complexes I and III (13–15, 38) and generate O_2_^·−^, and proximity to relatively high O_2_ levels, which accelerate O_2_^·−^ production (Fig. 8). Our findings indicate that ciliated cells overcome this challenge by expressing UCP2 and UCP5, which introduce a proton leak, dissipate the protonmotive force, decrease ROS production, and reduce lipid peroxidation. However, the proton leak partially uncouples respiration from ATP production. These findings indicate that ciliated cells sacrifice mitochondrial efficiency for safety from damaging oxidation.

Ciliated and goblet/secretory cells have specialized functions (39–41). Our data provide an additional distinction focused on their energy metabolism. First, mitochondria size differs, suggesting that function may differ (42). Second, ciliated cells express more UCP mRNA and protein. Third, although epithelia containing an abundance vs. a paucity of ciliated cells have the same mitochondrial O_2_ consumption, epithelia with few ciliated cells devote a greater share of mitochondrial respiration to generating ATP, i.e., coupled respiration. That result suggests that despite the local demand for ATP by beating cilia, ciliated cells may consume less ATP than goblet/secretory cells.^§^ We speculate that although ciliated cells will have a high local ATP production, goblet/secretory cells may have a higher total ATP production to support two energetically demanding processes, mucin and antimicrobial protein production and transepithelial electrolyte transport (41, 43–45).

UCP1 was the first uncoupling protein identified, and its role in short-circuiting the protonmotive force to generate heat in brown fat is well established (30, 46, 47). Subsequent studies found UCP2 relatively ubiquitously expressed, UCP3 expression largely restricted to skeletal muscle, and UCP4 and UCP5 predominately studied in the brain (30–32). These UCPs are much less abundant than UCP1, are not thought to be thermogenic, and are expressed in ectothermic animals (48, 49). Importantly, they decrease ROS production (50–58). Why ciliated airway epithelial cells express UCP2 and UCP5 rather than other UCPs is unknown. And why specifically UCP5 is predominantly expressed in ciliated cells vs. goblet/secretory and basal cells is puzzling. Comparison to other tissues emphasizes the uncertainty. For example, scRNAseq data from the Human Protein Atlas show that *UCP5* mRNA is more abundant in ciliated cells of the airway than in ciliated cells of the fallopian tube (*SI Appendix*, Fig. S4*A*). It is also notable that UCP5 is abundantly expressed in germ cells, which may be especially intolerant of oxidative injury to DNA (*SI Appendix*, Fig. S4*B*).

An even more interesting question is why did evolution use UCPs to protect airway ciliated cells against oxidant injury? The cost of this choice is significant because UCPs sacrifice mitochondrial efficiency in generating ATP. The alternative is to use antioxidants. Ciliated cells express mRNA for several antioxidant enzymes, and they almost certainly play a role. But why not run mitochondria at maximum efficiency and simply increase the amount of antioxidants to extinguish the ROS mitochondria churn out? A potential explanation relates to cellular geography. Strategically locating UCPs where O_2_^·−^ is generated offers a targeted solution; we expect that ROS-induced injury would be greatest locally, with mitochondria and cilia especially vulnerable. Consistent with that idea, when we knocked down UCPs, cilia beating slowed. Locally inhibiting O_2_^·−^ production would also minimize diffusion of highly reactive and potentially injurious ROS into the surrounding environment. On the other hand, elevating antioxidant levels to meet an intense local challenge carries its own risk; broadly decreasing ROS could interrupt important roles of ROS-mediated cell signaling and physiology. Thus, we speculate that UCPs located at the very site of ROS production might best meet an exceptional local threat driven by the geography of mitochondria position and a high O_2_ tension. On these points, Brand noted that decreasing ROS production with electron leak vs. decreasing ROS with antioxidants might be compared to prevention vs. cure (12). While the idea of prevention has logical appeal, how evolution weighs these two general strategies remains uncertain.

It may be informative to consider mitochondria in Himalayan Sherpas who have lived for 6,000 to 9,000 y at high altitudes. Their hypobaric hypoxia will decrease the contribution of O_2_ to ROS production (59–61), which may, in part, be responsible for findings opposite of those in airway ciliated cells, i.e., mitochondria in muscle from Himalayan Sherpas has decreased proton leak and increased coupled respiration. The increased mitochondrial efficiency may account for the decreased density of mitochondria in their muscle (62, 63). Further assessing adaptive responses to decreased vs. increased O_2_ at both the cellular and organ levels may yield understanding that triggers novel therapeutic approaches for humans challenged by hypoxia because of lung disease or hyperoxia because of treatment with inhaled O_2_.

This work has advantages and limitations. An advantage is that we used primary cultures of differentiated human airway epithelia at the air–liquid interface; they closely mimic in vivo human airway epithelia. In addition, the percentage of O_2_ in the cell culture incubator (18.5%)^†^ matches well with in vivo human data where humidified inspired air has 19.7% O_2_, with 5% CO_2_ it has 18.5% O_2_, and at the end of a complete slow expiration to residual volume, it can fall as low as 13.5% O_2_ (64). A limitation of the in vitro model may be lack of exposure to sheer stress/air flow, submucosal gland secretions, and disease-associated inflammatory changes that could alter airway ciliation and/or ROS generation. In addition, we studied large airway epithelia, and ciliated cells in distal airways might have different properties. An advantage is that we knocked down *UCP2* and *UCP5* after epithelia had already differentiated to avoid effects on development, and knocking down both *UCP2* and *UCP5* avoids compensation from the other UCP. A limitation is that UCP knockdown was not limited to ciliated cells. However, ciliated cells covered ~90% of the epithelia in those studies, suggesting that the major effects of knockdown result from changes in ciliated cells, and UCP5 is predominantly expressed in ciliated cells. A potential limitation is that we assessed changes in protonmotive force with TMRM, a fluorescent dye that accumulates in mitochondria and is used to assess mitochondrial membrane potential (65). However, TMRM is not a ratiometric reporter, and hence mitochondria abundance can influence fluorescence intensity. Although our analysis of human databases showed similar levels of mitochondrial protein transcripts across airway epithelial cell types, we cannot exclude the possibility that mitochondrial mass differed by cell type. Nevertheless, the impact of UCP2 and UCP5 knockdown on TMRM fluorescence in ciliated cells remains unaffected by this limitation. Here, we focused on intercellular ROS and did not investigate how it may contribute to extracellular ROS and its role in airway host defense. Another limitation is that we did not explore the regulation of UCP2 and UCP5 activity in airway epithelia. However, regulating UCP activity might be less important in a cell with a constant demand for ATP to fuel cilia than in cells with intermittent requirements. We are not aware of reports of UCP5 regulation, but UCP2 can be activated by O_2_^·−^ and 4-HNE, providing a potential negative-feedback mechanism that reduces ROS production (66–69).

Ciliated cells have an efficient supply chain. The producer of ATP (mitochondria) is located near the consumer (cilia) and an abundant supply (O_2_). But a byproduct (O_2_^·−^) of this organization poses the risk of injury. Our findings indicate that ciliated cells attenuate the risk by partially uncoupling respiration from energy production. Although this reduces the efficiency of oxidative phosphorylation, evolution must have favored this compromise to both power the cilia and minimize risk from local ROS toxicity.

## Materials and Methods

This study used primary cultures of differentiated human airway epithelia grown on permeable membrane supports. Airway epithelial cells were harvested from human lungs following protocols approved by the University of Iowa Institutional Review Board. In all cases, informed consent was obtained. Donor lungs were procured as postmortem specimens, as explants from patients undergoing lung transplant, or as lungs deemed not fit for transplant. Two methods were used to generate epithelia with different degrees of ciliation. Method 1 differentiated epithelial cells in USG *vs.* Pneumacult-ALI media. Method 2 differentiated epithelial cells in Pneumacult-ALI media in 0.5% O_2_ vs. 18.5% O_2_. All studies were done at 18.5% O_2_. Details on these and other methods are provided in *SI Appendix*, covering differentiated cultures of human airway epithelia, Pharmacologic interventions, Re-analysis of single cell RNA sequencing data, Transmission electron microscopy, Immunofluorescence of primary cultures of airway epithelia and human or pig lung tissue, Quantification of percent ciliation, Flow cytometry of differentiated culture of airway epithelia, Measurements using the CellROX-green fluorescent probe, Measurements of ESR using CMH, Measurement of rate of oxygen consumption rate using the Seahorse assay, RT-qPCR, Measurement of CBF, Antisense oligonucleotide-mediated knockdown of UCP2 and UCP5 in differentiated epithelia, Processing of epithelia samples for metabolic profiling, LC–MS method, GC–MS method, Metabolomics data analysis, and statistical analysis.

## Supplementary Material

Appendix 01 (PDF)

## Data Availability

All study data are included in the article and/or *SI Appendix*.

## Footnotes

^*^Although the rate at which beating airway cilia consume ATP is unknown, one can make a rough estimate based on published data about flagella, which share the basic structure of airway cilia (70, 71). A demembranated flagellum (~45 μm long) of a sea urchin sperm consumes ~2.3 × 10^5^ ATP molecules per beat (72). If we consider that a ciliated airway epithelial cell has cilia that are ~6 to 7 μm long, that there are ~200 to 300 cilia per cell, and that they beat at ~10 to 15 times/s, then one can estimate that the beating cilia would consume ~0.9 to 2.1 × 10^8^ ATP molecules/s (2, 73–75). The rate at which ciliated cells consume ATP is unknown, but several calculations estimate that an average human cell consumes ~1 × 10^7^ to 1 × 10^9^ ATP molecules/s (43). While the precision of these estimates is uncertain, they emphasize that ciliary activity likely consumes a major fraction of the ATP produced in ciliated airway epithelia.

^†^The percentage O_2_ for these experiments is calculated as 18.5% rather than 21% because of contributing partial pressures of CO_2_ and water vapor. Barometric pressure is 760 mm Hg. Water vapor pressure is 47 mm Hg. CO_2_ is 5%, which is 38 mm Hg (i.e., 0.05 × 760 = 38). The remaining partial pressure is 760 mm Hg − 38 mm Hg − 47 mm Hg = 675 mm Hg. 21% of 675 mm Hg = 141 mm Hg. Thus, 141 mm Hg/760 mm Hg = ~18.5% O_2_ in the cell culture incubator.

^‡^O_2_ in air will dissolve in the airway surface liquid (water) according to Henry’s law (76). The path to the mitochondria is through the airway surface liquid (~7 to 8 μm) and the distance from the apical membrane to the mitochondria (~2 μm). The O_2_ concentration at any point along that pathway will depend on the diffusion rate of O_2_ through the airway surface liquid and the cell, any effect of airway surface liquid stirring by cilia, and the rate of O_2_ consumption by the apical mitochondria. Although the absolute O_2_ concentration at mitochondria in epithelia residing at the air–liquid interface is unknown, it will be much higher than that in epithelia that are not air exposed; the path to reach apical mitochondria in that case would extend from red blood cells that carry O_2_ at a partial pressure that is ~2/3 that in humidified air, through endothelial cells, past other cells (e.g., fibroblasts) that consume O_2_, to the basement membrane, and then from the basement membrane to mitochondria just below the apical cell membrane. The difference in O_2_ delivery from air above epithelia vs. the tissue below the epithelia is also apparent from the observation that in most tissues O_2_ is 2 to 7% (18, 19).

^§^Basal cells will also contribute to O_2_ consumption. However, compared to more differentiated cells, stem and progenitor cells depend less on oxidative phosphorylation (77–79). Consistent with that, basal cells, which serve as stem cells in airway epithelia, have higher levels of transcripts for glycolytic enzymes (*SI Appendix*, Fig. S4*C*).
