# Supplementary material for: Mitochondrial uncoupling proteins protect human airway epithelial ciliated cells from oxidative damage
Source: Proc Natl Acad Sci U S A. 2024 Feb 28;121(10):e2318771121. doi: 10.1073/pnas.2318771121 (PMC10927548; doi:10.1073/pnas.2318771121)
Supplement: Supplementary file 1 — Appendix 01 (PDF) [file pnas.2318771121.sapp.pdf]

Supporting Information for

Mitochondrial uncoupling proteins protect human airway epithelial ciliated cells from oxidative damage

Akansha Jain, Bo Ram Kim, Wenjie Yu, Thomas O. Moninger, Philip H. Karp,
Brett A. Wagner, Michael J. Welsh

This PDF file includes:

Materials and methods
Figures S1 to S4
Supporting references

MATERIALS AND METHODS

Differentiated cultures of human airway epithelia

Primary cultures of airway epithelia were derived from cells harvested from multiple human donor airway tissues, as previously reported [1]. Briefly, proximal bronchi were dissected, cut into small pieces, and enzymatically digested. Epithelial cells were isolated and seeded onto collagen-coated inserts (Costar, 3470; Falcon, 353180) and allowed to differentiate at the air-liquid interface (ALI) for three weeks or more before the assays were done. We used two methods to generate epithelia with different degrees of ciliation. For Method 1, epithelial cells were differentiated using either UltroserG (USG) media or Pneumacult-ALI (PC-ALI) media to generate epithelia with few *vs.* abundant ciliated cells, respectively. For Method 2, epithelia were differentiated in Pneumacult-ALI (PC-ALI) media at two different O_2 levels; they were placed in sealed humidified chambers (STEMCELL Technologies, 27310) filled with either 18.5% O_2, 5% CO_2, remainder N_2 or 0.5% O_2/ 5% CO_2/ remainder N_2. All studies were performed at 18.5% O_2. Whenever feasible, studies followed a paired design so that epithelia from the same donor were assayed under both control and treatment conditions.

Pharmacologic interventions

The following pharmacologic reagents were used: oligomycin (Sigma-Aldrich, O4876), FCCP (Sigma-Aldrich, C2920), rotenone (Sigma-Aldrich, R8875), antimycin A (Sigma-Aldrich, A8674), and CMH (Enzo Life Sciences, ALX-430-117).

Re-analysis of single cell RNA sequencing data

We used three public single cell RNA sequencing datasets. The Goldfarbmuren et al., dataset obtained data from cells obtained directly from large airway tissue of six never smokers and six heavy smokers. Our analysis contains data only from the six never smokers [2]. The Deprez et al., dataset contains data from cells obtained from biopsies and brushings of large airways of ten healthy donors and our analysis contains all ten healthy donors [3]. In the Deprez et al. dataset, the suprabasal, secretory, and multiciliated clusters each comprised a subcluster of cells that could only be detected in nasal samples and were labeled as “Suprabasal N,” “Secretory N,” and “Multiciliated N” [3]. The Thurman et al., dataset contains five wild-type and five cystic fibrosis piglets and our analysis contains only the five wild-type piglets [4].

Transmission electron microscopy

Small pieces of freshly excised pig trachea from newborn pigs were fixed in 2.5% glutaraldehyde and post-fixed in 2% osmium tetroxide. Following en bloc staining with 2.5% uranyl acetate the tissues were dehydrated in acetone, infiltrated with Spurr’s epoxy resin and cured. 70 nm sections were cut and counterstained with 5% uranyl acetate and Reynold’s lead citrate. The sections were imaged in a Hitachi HT7800 equipped with and AMT camera.

Immunofluorescence of primary cultures of airway epithelia and human or pig lung tissue

Airway epithelia were washed three times with phosphate-buffered saline (PBS), fixed with 4% paraformaldehyde for one hour, rinsed three times with PBS-/-, and then permeabilized with 0.3% Triton-X for 20 minutes. To minimize nonspecific staining, epithelia were treated with SuperBlock (Thermo Fisher Scientific) containing 10% normal goat serum for one hour at room temperature. Primary antibodies were diluted in SuperBlock with 10% normal goat serum, and were added apically overnight at 4°C. After overnight incubation, epithelia were washed three

times with PBS-/- and incubated for one hour with appropriate secondary antibodies diluted in PBS. The following primary antibodies were used for immunofluorescence staining: rabbit anti-UCP2 (1:100; Proteintech cat. no. 11081) [5, 6]; rabbit anti-UCP5 (1:100; Novus Biologicals cat. no. NBP2-56930) [7]; rabbit anti-acetyl α-tubulin (1:100, Cell Signaling Technologies cat. no. 5335S) [8]; and mouse or rabbit anti-TOM70 (1:100; Proteintech cat. no. 14528-1-AP) [9, 10]. The following secondary antibodies were used: goat anti-rabbit conjugated to Alexa Flour 488 (1:1000; Thermo Fisher Scientific cat. no. A11070); and goat anti-mouse conjugated to Alexa Flour 568 (1:1000; Thermo Fisher Scientific cat. no. A11019). The actin cytoskeleton was stained with Alexa Fluor 633 phalloidin (1:5000; Thermo Fisher Scientific cat. no. A22284), which was added at the same time as the secondary antibodies. The epithelia were separated from plastic transwells by cutting the edges of the transwell with a razor blade. Epithelia were mounted on glass slides using Vectashield with DAPI (4',6'- diamidino-2-phenylindole; Vector Laboratories) and coverslips were sealed with clear nailpolish. Imaging was performed on an Olympus Fluoview FV 3000 confocal microscope or a Zeiss880 confocal microscope. Z-stack images were processed using the Olympus Fluoview program. Scale bars were added and images were processed using FIJI software.

Western blotting

Epithelia on filters were cut from transwells and lysed on ice in a 1.5 mL Eppendorf tubes with lysis buffer (5 mM EDTA, 1% NP40, 0.5% sodium deoxycholate) containing protease and phosphatase inhibitors. Cell lysate was centrifuged at 11,000 rpm for 10 min and supernatant was separated from pellet and filter. 4x Laemmli sample buffer (BioRad, 1610747) was added to the samples. Samples were loaded onto gels and protein was transferred onto PVDF membranes after activating with methanol using the trans-blot Turbo transfer system following the manufacturer's instructions (BioRad, 1704150). Antibodies used for immunoblots were as following, rabbit anti-acetyl α-tubulin (1:100, Cell Signaling Technologies cat. no. 5335S), total OXPHOS Rodent WB Antibody Cocktail (1:100, Abcam cat. no. ab110413), and rabbit anti-UCP5 (1:100, Proteintech cat. no. 25223-1-AP).

Quantification of percent ciliation

Maximum intensity projections of β-tubulin IV staining were used to analyze percent ciliation. We used ImageJ to subtract background fluorescence. Any intensity above that value was considered positive for ciliation.

Flow cytometry of differentiated culture of airway epithelia

Both the apical and basolateral compartments of the transwells were washed quickly with PBS-/- and incubated with 0.25% trypsin/EDTA (Gibco, 25200056). The cultures were incubated in this solution for 15 min at 37°C, after which the trypsin was neutralized with equal volume of PC-ALI medium. Single-cell suspensions were collected from cultured epithelia and spun at 250 x g for 3 min and washed twice with PBS-/-. All centrifuge spins were done at 250xg for 3 min. Live cells were filtered through a 100 mm cell strainer to remove any large aggregates in the samples. Live cells were incubated in 50 mL PBS-/- with 2% FBS containing 20 nM TMRM dye (Invitrogen, M20036),10 μg/mL DAPI, and conjugated primary antibodies. DAPI was used to separate live versus dead cells, ACE2 conjugated to 647 (Santa Cruz Biotechnologies, sc-390851) was used to identify specifically ciliated cells in the whole cell population, and TMRM dye was used to measure mitochondrial membrane potentials. FCCP (50 μM) was used as a

positive control for TMRM measurements. Data were collected using a flow cytometer (Attune Nxt, Invitrogen) and analyzed using the Flowjo software.

Measurements using the CellROX-green fluorescent probe
CellROX-green confocal imaging was performed on live cultures of airway epithelia. The epithelia were incubated in 2.5 μM CellROX-green Reagent and 5 μg/mL Hoechst 33342 diluted in PBS-/- (applied both apically and basolaterally) for 30 minutes at 37 °C, 5% CO_2. They were then rinsed three times with PBS-/-. Live cell imaging of CellROX-green was quickly performed on a Zeiss880 confocal microscope. At least three fields were captured per epithelia. For each field, the integrated densities of fluorescence were obtained, and background was subtracted using a threshold on the FIJI software.

Measurements of electron spin resonance (ESR) using CMH
Levels of free radicals were measured using electron spin resonance (ESR) spectroscopy as previously described [11-13]. Briefly, 1-Hydroxy-3-methoxycarbonyl-2,2,5,5-tetramethylpyrrolidine (CMH) (Enzo Life Sciences, Inc., Farmington, NY, USA) was prepared in PBS containing 100 μM of diethylenetriaminepentaacetic acid (DETAPAC) under an argon flow. Airway epithelial cultures on transwell membranes were incubated for one minute in 250 μM CMH. The membrane was then twice washed quickly with PBS-/-, twice, and placed into tubing which was then centered into a Bruker ERO 4119 HS cavity. Spectra were obtained with a Bruker EMX X-band spectrometer. The spectrometer parameters were: centerfield 3502 G; sweep width 61 G; microwave frequency 9.85 GHz; microwave power 10 mW; receiver gain 1×10^5; modulation frequency 100 kHz; modulation amplitude 1 G. The signal amplitude was determined using the Bruker WinEPR acquisition software (Billerica, MA).

Measurement of rate of oxygen consumption rate using the seahorse assay
The mitochondrial oxygen consumption rate (OCR) was measured in live cultures of airway epithelia. Epithelia were excised from transwells using 3 mm biopsy punches, with one punch produced per transwell. The biopsy punches were carefully loaded into Seahorse XFe24 iselt microplates, and 0.5 mL/well Seahorse XF Dulbecco's Modified Eagle's Medium (DMEM) supplemented with 2 mM L-glutamine, 15.5 mM glucose, and 1 mM sodium pyruvate (pH 7.4). Epithelia biopsies were washed two times with Seahorse XF DMEM. Epithelia biopsy punches were acclimated for 1 hour at 37 °C without CO_2 and transferred to a Seahorse XFe24 analyzer for analysis. All OCR measurements were performed using a 3–2–3-min cycle of mix–wait–measure periods. Final concentrations of oligomycin, FCCP, rotenone and antimycin A in the wells were 3 μM, 0.5 μM, 1 μM, and 1 μM, respectively. The optimal FCCP concentration was determined by titrating FCCP; the concentration that resulted in the largest increase in OCR was used for further experiments.

Reverse transcriptase quantitative polymerase chain reaction (RT-qPCR)
Total RNA was isolated from samples using either the RNeasy Mini Kit (Qiagen, Inc. 74104) or the RNeasy Micro Kit (Qiagen, Inc. 74004). Genomic DNA was removed using DNase I (QIAGEN). RNA quality was verified using a NanoDrop 2000 spectrophotometer (Thermo Fisher Scientific), and samples with a 260:280 ratio ≥ 1.8 were carried forward. RNA was reverse transcribed using the SuperScript VILO MasterMix (Invitrogen) or High-Capacity cDNA Reverse Transcription kit (Applied Biosystems). Amplification was performed using gene-

specific primers and the Fast SYBR Green Master Mix (Applied Biosystems) on the QuantStudio6Pro Real-Time PCR System (Applied Biosystems). The housekeeping gene was *TATA-box binding protein* (*TBP*). All primers were designed and validated using a melting curve. *UCP2* and *UCP5* versus *FOXJ1* correlation plots show negative delta Ct values on x- and y-axes (delta Ct =gene of interest Ct value minus housekeeping gene Ct value). For RT-qPCR data shown in Figures 4A-4C, the epithelial cells were grown under two methods to alter number of ciliated cells (Method 1: USG versus PC-ALI media both grown at 18.5% and Method 2: differentiation in PC-ALI media under 0.5% or 18.5% O_2).

Primers are as follows:
UCP2-Forward: AGTCCGGTTACAGATCCAAGG;
Reverse: AGCCCATTGTAGAGGCTTCG
UCP5-Forward: TTTTAACTCACTTCGTTTCCAGC;
Reverse: CGATTGCCCTCTGGTTCATCA
FOXJ1-Forward: CCCACCTGGCAGAATTCAATCCG;
Reverse: CTCAGTAGCCGCTCCGCGTAC
TBP- Forward: TGTGCACAGGAGCCAAGAGT;
Reverse: ATTTTCTTGCTGCCAGTCTGG
ND1-
Forward: CCACCTCTAGCCTAGCCGTTTA
Reverse: GGGTCATGATGGCAGGAGTAAT
ND6-
Forward: CAAACAATGTTCAACCAGTAACCACTAC
Reverse: ATATACTACAGCGATGGCTATTGAGGA

Measurement of ciliary beat frequency
Phase-contrast videos of ciliary motion were obtained using the Zeiss Axio Observer microscope at 50 frames per second. For each field, 256 frames (5.1 second videos) were obtained. A minimum of three random fields per culture were measured. The whole-field gaussian mean ciliary beat frequencies (CBF) was analyzed using the Sisson-Ammons video analysis software (SAVA; Ammons Engineering, Mt. Morris, MI). CBF measurements were obtained at room temperature (20° C).

Antisense oligonucleotide-mediated knockdown of UCP2 and UCP5 in differentiated epithelia
Antisense oligonucleotides (ASOs) were synthesized by Integrated DNA Technologies Inc. (Coralville, IA USA) as previously described [14] and used to knock down UCP2 and UCP5 in airway epithelial cultures. The sequences of the ASOs are non-targeting (NT) control, +G+G+CTACTACGCCG+T+C+A [15]; UCP5, +T+A+TCGATAAAGCT+T+C+C for UCP5; and UCP2, +C+G+AGCAACATTGG+G+A+G. The phosphodiester backbone was replaced with a phosphorothioate backbone and the + sign indicates an Affinity Plus locked nucleic acid base [16]. An ASO stock (10 mM) was prepared in TE-buffer (10 mM Tris, pH 7.5 or 8.0, 0.1 mM EDTA) and diluted to a final concentration of 10 μM in PC-ALI medium (STEMCELL Technologies). Cultures were fed ASO-containing medium every three days over the course of twelve days. At day 12 post-ASO treatment, the cultures were harvested for use in downstream

assays. All knockdown experiments were performed on epithelia differentiated in PC-ALI media.

Processing of epithelia samples for metabolic profiling

Epithelia cultures were washed twice with ice-cold PBS, then twice with ice-cold water. The inserts were flash frozen in liquid nitrogen and then lyophilized. Each transwell received 1.2 ml of extraction buffer (with internal standards D4-citric acid, D4-succinic acid, D8-valine, and U13C-labeled glutamine, glutamic acid, lysine, methionine, serine, and tryptophan; Cambridge Isotope Laboratories) and the cells were scraped off the membrane and transferred to a 1.5 ml microcentrifuge tube. The samples were frozen in liquid nitrogen, sonicated for 10 minutes, and transferred to a -20° C freezer where they were rotated for 1 hour. The samples were then centrifuged at 21,000xg for 10 minutes and the supernatants were transferred to new 1.5 ml microcentrifuge tubes. Each supernatant was split into 300 µl aliquots that were transferred to a microcentrifuge tube for LC-MS. The extracts were dried using a speed vac apparatus and used for LC-MS.

LC-MS method

Dried extracts were reconstituted in 30 µL acetonitrile/water (1:1 v/v), vortexed well, rotated on a rotator at -20C overnight, and centrifuged. The supernatant was then transferred to LC-MS autosampler vials for analysis. LC-MS data were acquired on a Thermo Q Exactive hybrid quadrupole Orbitrap mass spectrometer with a Vanquish Flex UHPLC system or a Vanquish Horizon UHPLC system. The LC column used was a Millipore SeQuant ZIC-pHILIC (2.1 X 150 mm, 5 µm particle size) with a ZIC-pHILIC guard column (20 x 2.1 mm). The injection volume was 2 µL. Mobile phase is as follows: Solvent A was 20 mM ammonium carbonate [(NH4)2CO3] and 0.1% ammonium hydroxide (v/v) [NH_4OH] [Note: pH was ~9.1] and solvent B was acetonitrile. Samples were run at a flow rate of 0.150 mL/min. The gradient started at 80% Solvent B and decreased to 20% B over 20 minutes; it was then returned to 80% B over 0.5 minutes and held there for 7 minutes.

Metabolomics data analysis

Acquired LC-MS data were processed using the Thermo Scientific TraceFinder 4.1 software, and metabolites were identified based on the University of Iowa Metabolomics Core facility standard-confirmed, inhouse library. The Normalization and Evaluation of Metabolomics Data (NOREVA) database was used to correct for signal drift [17]. The NOREVA-corrected data were then normalized to the sum total of signal per sample to control for extraction, derivatization, and/or loading effects.

Statistical analysis

Statistical analysis of treatment versus non-treatment groups was completed using a two-tailed, student's t-test for two groups or an ANOVA for >3 groups. When data were paired, a paired t-test was performed. Experimental data are represented as mean ± SEM.

SUPPLEMENTAL FIGURES

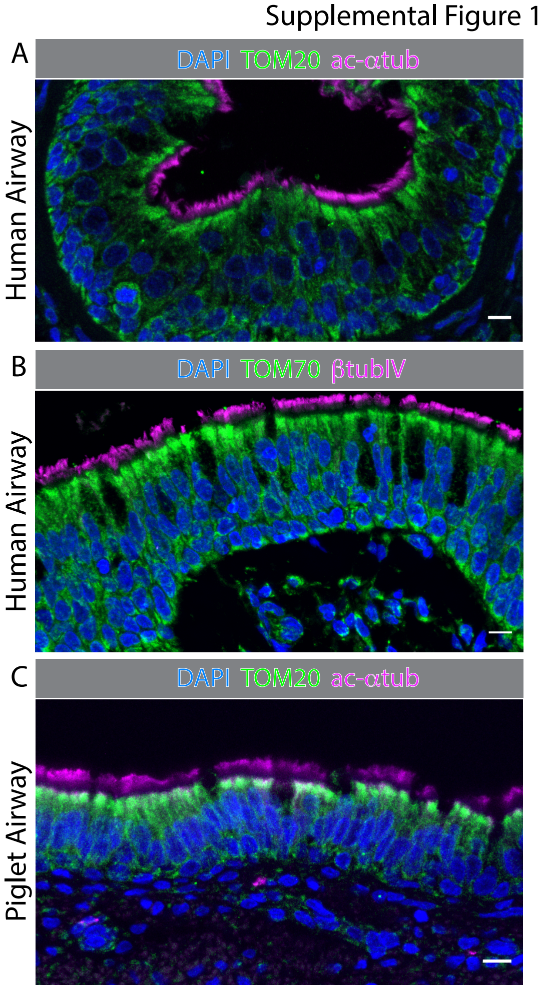

Figure S1. Immunofluorescence images show apical localization of mitochondria in ciliated airway epithelial cells.

Images are immunofluorescence staining of large airway tissue. Acetylated α-tubulin marks cilia (magenta), and DAPI marks nuclei (blue) in all images. Scale bars indicate 10 μm. **(A)** TOM20 immunostaining marks mitochondria (green) in human airway epithelia. **(B)** TOM70 immunostaining marks mitochondria (green) in human airway epithelia. **(C)** TOM20 immunostaining marks mitochondria (green) in pig airway epithelia.

Supplemental Figure 2

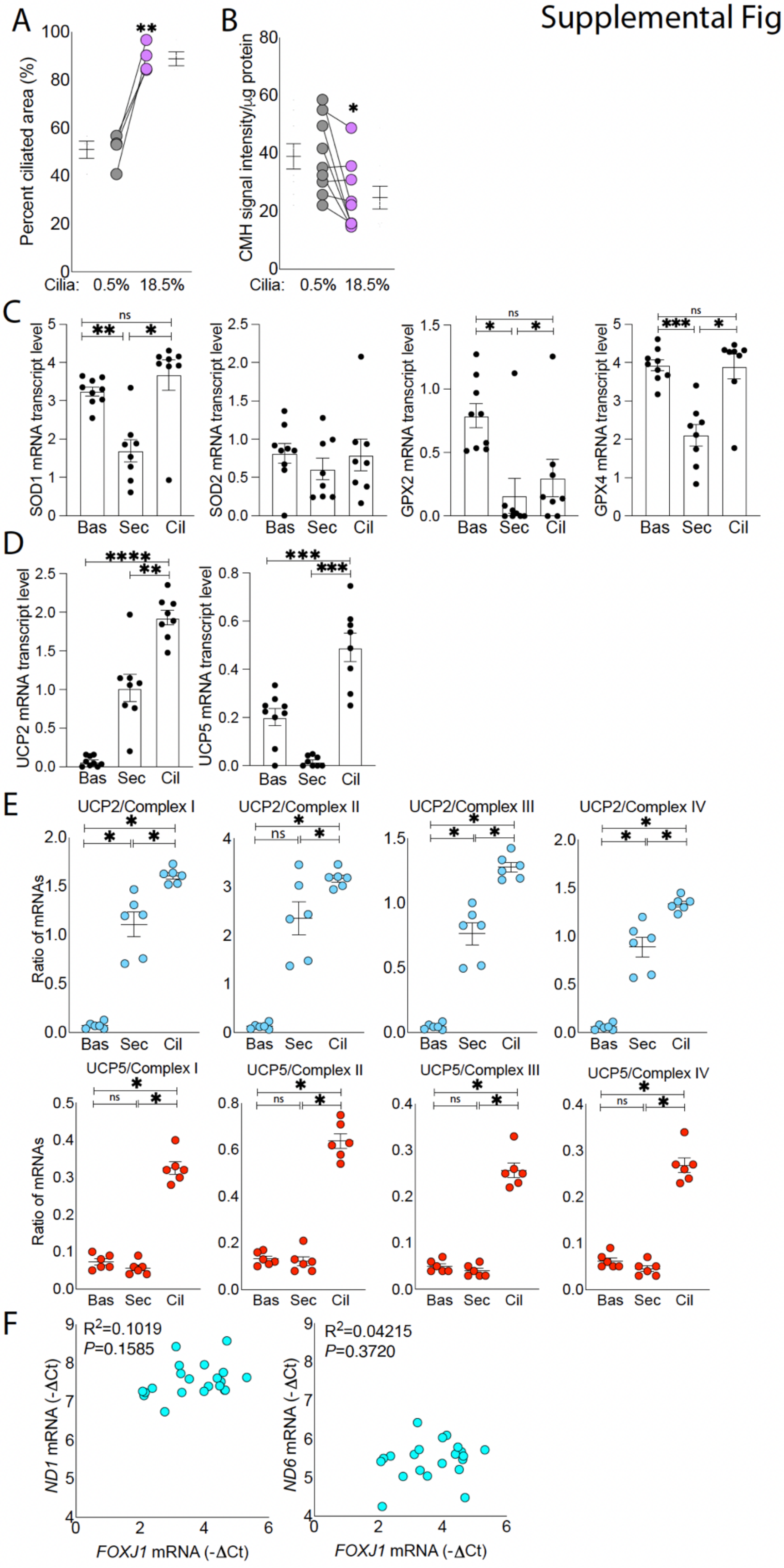

Figure S2. Intracellular ROS levels and scRNA-seq data for antioxidants and *UCP2* and *UCP5*. **(A)** Qualification of percentage of airway surface covered by cilia determined by immunostaining with β-tubulin IV in airway cells differentiated in 0.5% or 18.5% O_2 (Method 2). **(B)** Level of ROS signal intensity normalized to protein measured using ESR.

For panels A and B, each set of data points and lines is from a different human donor. Bars and whiskers indicate mean ± SEM. ** indicates P<0.01 and * indicates P<0.05 by paired Student's *t* test. **(C)** mRNA transcript levels of multiple antioxidant enzymes in basal, secretory, and ciliated cells of the proximal airway. Data are from a public single-cell database of the human large airways [3]. **(D)** Transcript levels of *UCP2* and *UCP5* mRNA in basal, secretory, and ciliated cells of the proximal airway [3]. **(E)** Data from Goldfarbmuren et al. database showing *UCP2* and *UCP5* mRNA divided by average mRNA of multiple different ETC complexes (complex I, complex II, complex III, and complex IV) [2]. **(F)** mRNA levels for the mitochondrial proteins ND1 and ND6 *vs. FOXJ1* mRNA levels in airway epithelia differentiated in USG at 18.5% O_2. For panels C-F, the asterisks indicate ***P<0.001, **P<0.01, *P<0.05 by ANOVA.

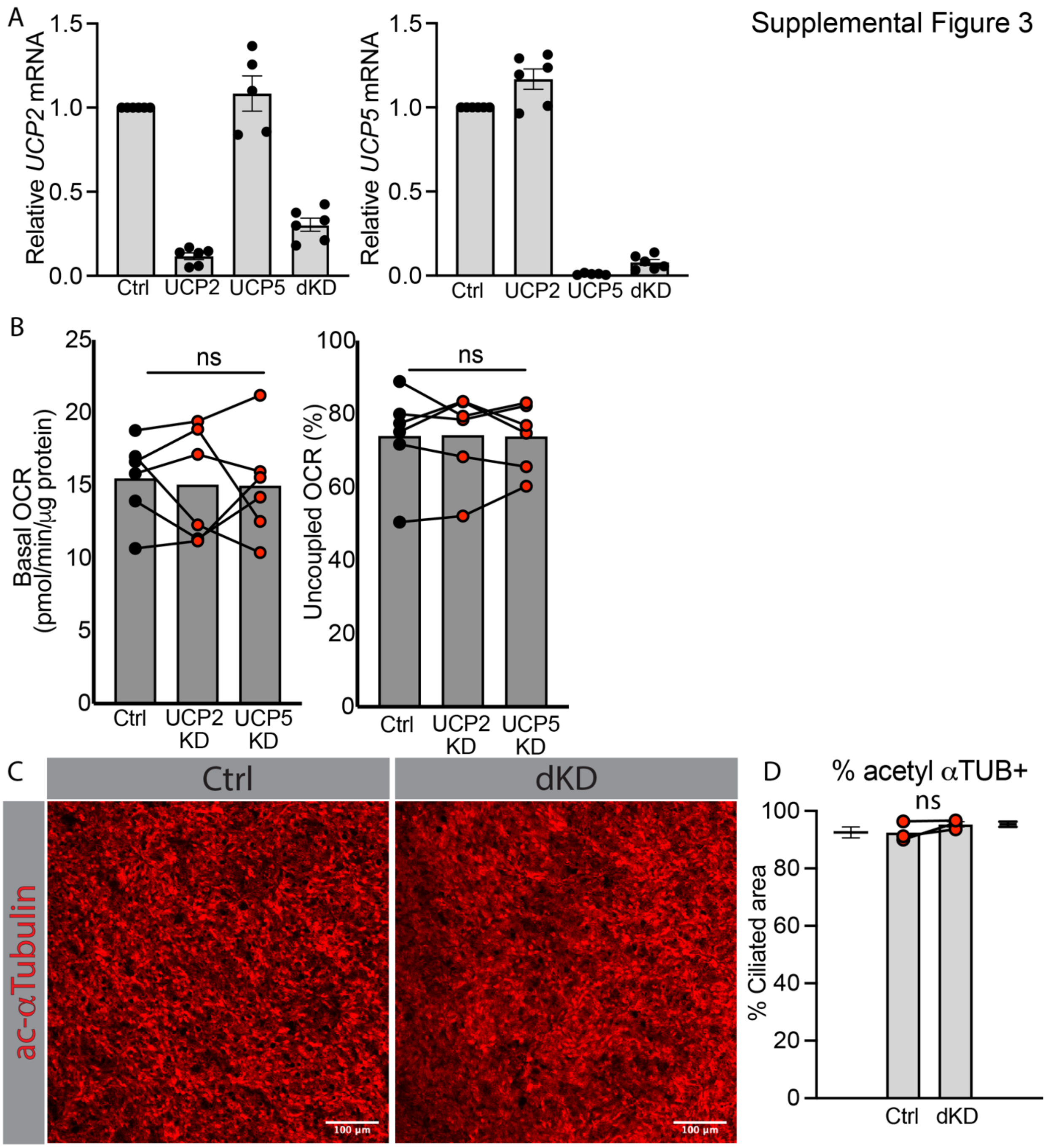

Figure S3. Antisense oligonucleotide knockdown of *UCP2* and *UCP5* mRNA. **(A)** Knockdown of individual *UCP2* or *UCP5* antisense oligonucleotide resulted in a small increase in mRNA levels of the other uncoupling protein. **(B)** Basal respiration and uncoupled respiration (as a percentage of total respiration) in epithelia with either *UCP2* or *UCP5* knockdown. **(C)** Immunofluorescence images of acetylated α-tubulin, which marks cilia (red), in Ctrl and *UCP2* and *UCP5* double knockdown (dKD) treated epithelia. **(D)** Percentage of airway epithelial surface positive for acetylated α-tubulin immunostaining in Ctrl vs. dKD (*UCP2* and *UCP5*) treated epithelia.

Supplemental Figure 4

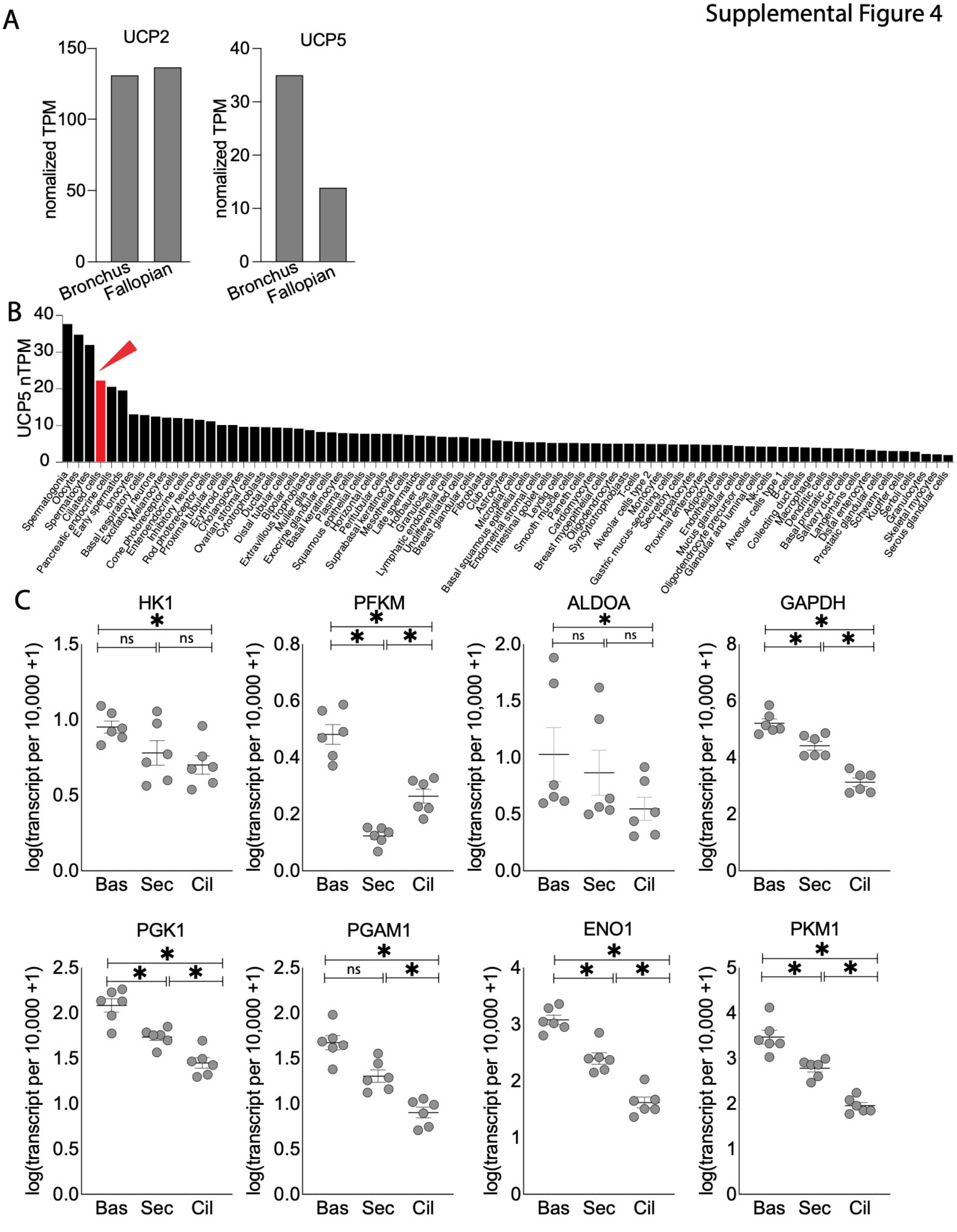

Figure S4. Transcript levels for UCPs and glycolytic enzymes in various cell types. **(A)** *UCP2* and *UCP5* mRNA levels in ciliated cells of bronchus and fallopian tube. Normalized TPM shown as determined by the Human Protein Atlas version 23.0 database [18, 19]. (**B)** Normalized transcripts per million (nTPM) scRNA-seq data for *UCP5* across cell types from different tissues. Data were obtained from the Human Protein Atlas version 23.0 database [18, 19]. **(C)** mRNA levels of multiple glycolytic enzymes in basal (Bas), secretory (Sec), and ciliated (Cil) cells of human large airways obtained from a public scRNA-seq database [2]. HK1: Hexokinase 1; PFKM: phosphofructokinase, muscle; ALDOA: aldolase A; GAPDH: glyceraldehyde-3-phosphate dehydrogenase; PGK1: phosphoglycerate kinase 1; PGAM1: phosphoglycerate mutase 1; ENO1: enolase 1; PKM1: pyruvate kinase M1. Each data point is from a different human donor. Bars and whiskers indicate mean ± SEM. Asterisk indicates $P<0.05$ by ANOVA.

SUPPORTING REFERENCES

1. Karp, P.H., et al., *An in vitro model of differentiated human airway epithelia. Methods for establishing primary cultures.* Methods Mol Biol, 2002. **188**: p. 115-37.
2. Goldfarbmuren, K.C., et al., *Dissecting the cellular specificity of smoking effects and reconstructing lineages in the human airway epithelium.* Nat Commun, 2020. **11**(1): p. 2485.
3. Deprez, M., et al., *A Single-Cell Atlas of the Human Healthy Airways.* Am J Respir Crit Care Med, 2020. **202**(12): p. 1636-1645.
4. Thurman, A.L., J.A. Ratcliff, M.S. Chimenti, and A.A. Pezzulo, *Differential gene expression analysis for multi-subject single-cell RNA-sequencing studies with aggregateBioVar.* Bioinformatics, 2021. **37**(19): p. 3243-3251.
5. Yu, S.X., et al., *Genipin inhibits NLRP3 and NLRC4 inflammasome activation via autophagy suppression.* Sci Rep, 2015. **5**: p. 17935.
6. Ji, F., T. Shen, W. Zou, and J. Jiao, *UCP2 Regulates Embryonic Neurogenesis via ROS-Mediated Yap Alternation in the Developing Neocortex.* Stem Cells, 2017. **35**(6): p. 1479-1492.
7. Ferrer, I., et al., *Familial globular glial tauopathy linked to MAPT mutations: molecular neuropathology and seeding capacity of a prototypical mixed neuronal and glial tauopathy.* Acta Neuropathol, 2020. **139**(4): p. 735-771.
8. Mao, S., et al., *Motile cilia of human airway epithelia contain hedgehog signaling components that mediate noncanonical hedgehog signaling.* Proc Natl Acad Sci U S A, 2018. **115**(6): p. 1370-1375.
9. Konig, T., et al., *MIROs and DRP1 drive mitochondrial-derived vesicle biogenesis and promote quality control.* Nat Cell Biol, 2021. **23**(12): p. 1271-1286.
10. Xu, X., et al., *Sp1 promotes tumour progression by remodelling the mitochondrial network in cervical cancer.* J Transl Med, 2023. **21**(1): p. 307.
11. Dikalov, S., B. Fink, M. Skatchkov, and E. Bassenge, *Comparison of glyceryl trinitrate-induced with pentaerythrityl tetranitrate-induced in vivo formation of superoxide radicals: effect of vitamin C.* Free Radic Biol Med, 1999. **27**(1-2): p. 170-6.
12. Dikalov, S., M. Skatchkov, B. Fink, and E. Bassenge, *Quantification of superoxide radicals and peroxynitrite in vascular cells using oxidation of sterically hindered hydroxylamines and electron spin resonance.* Nitric Oxide, 1997. **1**(5): p. 423-31.
13. Dikalov, S.I., A.E. Dikalova, and R.P. Mason, *Noninvasive diagnostic tool for inflammation-induced oxidative stress using electron spin resonance spectroscopy and an extracellular cyclic hydroxylamine.* Arch Biochem Biophys, 2002. **402**(2): p. 218-26.
14. Alharbi, A.S., et al., *Rational design of antisense oligonucleotides modulating the activity of TLR7/8 agonists.* Nucleic Acids Res, 2020. **48**(13): p. 7052-7065.
15. Linnane, E., et al., *Differential uptake, kinetics and mechanisms of intracellular trafficking of next-generation antisense oligonucleotides across human cancer cell lines.* Nucleic Acids Res, 2019. **47**(9): p. 4375-4392.
16. Anderson, B.A., et al., *Towards next generation antisense oligonucleotides: mesylphosphoramidate modification improves therapeutic index and duration of effect of gapmer antisense oligonucleotides.* Nucleic Acids Res, 2021. **49**(16): p. 9026-9041.
17. Li, B., et al., *NOREVA: normalization and evaluation of MS-based metabolomics data.* Nucleic Acids Res, 2017. **45**(W1): p. W162-W170.

18. Karlsson, M., et al., *A single-cell type transcriptomics map of human tissues.* Sci Adv, 2021. **7**(31).
19. *The human protein atlas*. Available from: https://www.proteinatlas.org/
